# Dihydromyricetin and Neuroinflammation: Implications for CNS Disorders and Therapeutic Development

**DOI:** 10.3390/ijms27209080

**Published:** 2026-10-13

**Authors:** Samantha G. Skinner, Eliana G. Aleman, Ashley N. Berges, Katie J. Kim, Dhruv Kantilal, Liana Asatryan, Daryl L. Davies

**Affiliations:** Titus Family Department of Clinical Pharmacy, University of Southern California Alfred E. Mann School of Pharmacy and Pharmaceutical Sciences, Los Angeles, CA 90033, USA

**Keywords:** dihydromyricetin, neuroinflammation, central nervous system, NF-κB, NLRP3 inflammasome, autophagy, oxidative stress, neurodegenerative diseases, stroke

## Abstract

Dihydromyricetin (DHM) is a bioactive polyphenol with broad therapeutic potential, including strong anti-inflammatory activity. Importantly, DHM has been shown to cross the blood–brain barrier (BBB), enabling pharmacological activity within the central nervous system (CNS) and positioning it as a candidate for CNS disorders in which neuroinflammation plays a central role, including Alzheimer’s disease, Parkinson’s disease, and stroke. Therapeutics such as DHM, which address neuroinflammation, are of increasing interest. Across CNS disease models, DHM’s effects show recurring convergence on NF-κB suppression, NLRP3 inflammasome inhibition, and autophagy regulation, alongside context-specific mechanisms involving oxidative stress, ferroptosis, neurotrophic signaling, and peripheral gut–CNS communication. This review examines the evidence for DHM as a neuroinflammatory modulator, focusing on the mechanisms by which it exerts its therapeutic effects. By doing so, we aim to support its translational potential as a natural product for improving human health.

## 1. Introduction

According to the World Health Organization, more than one in three people worldwide are affected by a neurological condition, making these disorders the leading cause of death and disability globally [1]. Neuroinflammation is a common feature of many central nervous system (CNS) pathologies, which include neurodegenerative diseases, neuropsychiatric disorders, and conditions related to CNS injury or cerebrovascular diseases. While inflammation plays a critical role in maintaining CNS homeostasis and responding to injury, persistent or dysregulated activation can drive pathological processes and contribute to CNS disease progression. This inappropriate response is mediated by a complex network of molecular signals and cellular responses, including glial cell activation, blood–brain barrier (BBB) dysfunction, and peripheral immune cell infiltration. These pathological processes have been closely linked to symptom exacerbation and CNS degeneration in diseases such as Alzheimer’s, Parkinson’s, and ischemic stroke [2,3,4].

Despite the critical role of neuroinflammation in CNS diseases, few FDA-approved therapeutics specifically target this process, reflecting in part the complexity of neuroinflammatory signaling, which lacks a singular cellular target. Efforts to identify therapeutic agents therefore encompass novel chemical development, drug repurposing, and the exploration of natural products, including phenolic compounds. Polyphenols are a class of bioactive compounds abundant in human nutrition that exhibit remarkable pleiotropy, engaging a host of protein and lipid targets implicated in inflammation [5]. Given this activity, nutraceutical compounds in this class have been rigorously investigated for their therapeutic effects in a variety of conditions, including cardiovascular diseases, cancers, metabolic disorders, and infection [6,7,8,9]. Their potent anti-inflammatory properties also make them attractive candidates for modulating neuroinflammation.

Dihydromyricetin (DHM) is a bioactive polyphenolic compound found in several plant species used in Traditional Chinese Medicine, including *Ampelopsis grossedentata*, *Hovenia dulcis*, and *Cedrus deodara* [10]. The compound exhibits potent anti-inflammatory properties in both peripheral and central tissues, and exhibits favorable preclinical tolerability [11,12]. Preclinical evidence indicates that DHM reaches brain tissue following systemic administration. In rats, DHM was detected in brain tissue for up to 12 h following oral administration at 100 mg/kg [13]. In mice administered 50 mg/kg DHM, whole-brain concentrations measured 15 min after dosing were 50.9 ± 2.6 and 423 ± 66 nmol/g following oral administration and 864 ± 220 and 672 ± 85 nmol/g following intraperitoneal administration in female and male mice, respectively [14]. DHM was not detected in brain tissue at 45 min or later, although trace amounts of the metabolites 3′-O-methyl-DHM and 4′-O-methyl-DHM were detected 15 min after intraperitoneal administration. Together with its reported effects on mitochondrial function, autophagy, apoptosis, and inflammatory signaling, this evidence has prompted investigation of DHM as a potential therapeutic candidate for several CNS conditions [15,16]. However, much remains to be understood regarding DHM’s mechanism of therapeutic action in the brain and CNS. This narrative review examines recent literature on DHM’s neuroinflammatory mechanisms, focusing on the molecular mechanisms by which DHM alleviates neuroinflammation. In doing so, we aim to provide support for the translational potential of DHM for CNS conditions.

A recent review summarized the broader pharmacological profile of DHM across central nervous system and peripheral disease contexts, including antioxidant, anti-apoptotic, and metabolic effects [16]. Here, we narrow the scope specifically to neuroinflammatory mechanisms, applying explicit inclusion and exclusion criteria to studies in which an inflammatory pathway is the primary or parallel driver of the reported effect. The disease contexts discussed in this narrative review represent the CNS conditions identified in the available literature in which DHM’s effects have been linked to the modulation of neuroinflammatory processes. Across identified studies, emerging evidence points to recurring modulation of an NF-κB/NLRP3/autophagy inflammatory axis across otherwise distinct CNS disorders, suggesting potential mechanistic convergence beyond disease-specific pathways. We further extend coverage to disease contexts and mechanisms not addressed in that prior review, including spinal cord injury and gut-microbiome-mediated peripheral signaling, and incorporate literature published subsequent to it.

## 2. Anti-Inflammatory Action of DHM in CNS

Given the role of neuroinflammation in CNS disease progression, therapeutic agents that can attenuate inflammatory signaling represent promising modalities for intervention. DHM, based on its demonstrated ability to suppress pro-inflammatory cytokines, inhibit glial activation, and downregulate key inflammatory signaling pathways, has emerged as a promising candidate. In this section, we review experimental studies that investigate DHM’s capacity to modulate neuroinflammatory responses in both in vitro and in vivo models. Table 1 summarizes the current body of evidence supporting DHM’s anti-inflammatory action in the CNS across multiple disease contexts. Detailed experimental characteristics of the included studies are provided in Supplementary Tables S1 and S2, which summarize the in vitro exposure conditions and in vivo dosing regimens, respectively.

### 2.1. Neurodegenerative Disorders

#### 2.1.1. Alzheimer’s Disease

Alzheimer’s Disease (AD) is a debilitating neurodegenerative disease marked by distinct pathological features, including the formation of amyloid beta (Aβ) plaques, neurofibrillary tangles, and tau deposits [45]. While the mechanisms driving disease onset and progression are still unclear, neuroinflammation is widely accepted as a key driver of AD pathophysiology, as activation of microglia and astrocytes, release of inflammatory cytokines, and Aβ and tau pathologies all contribute to neuronal death and brain atrophy [46,47]. DHM has been investigated across in vitro and in vivo AD models for its anti-inflammatory, anti-amyloidogenic, and signaling-modulatory effects.

At the microglial level, DHM’s anti-inflammatory effect traces to direct inhibition of TLR4 signaling. Pei et al. (2023) demonstrated that DHM binds myeloid differentiation protein MD2, blocking TLR4 complexation and thereby reducing microglial activation, apoptosis, and IL-1β, IL-6, and TNF-α levels in BV2 cells, with corresponding reductions in TLR4 expression and cognitive improvement in APP/PS1 mice [17]. This cytokine-suppressing effect is not model-specific: Wang et al. (2020) found that DHM prevents hippocampal elevation of IL-1β, IL-6, and TNF-α following intracerebroventricular Aβ infusion in Sprague-Dawley rats [20]. Beyond cytokine suppression, Sun et al. (2019) linked the same downstream cytokine profile to restoration of dysregulated AMPK/SIRT signaling in AD, a pathway critical to cellular homeostasis, autophagy, and neuroinflammation [19,48]. DHM activates this pathway to exert a neuroprotective effect, normalizing levels of p-AMPK/AMPK, Bcl-2, Bax, and NF-κB p65 alongside cognitive gains.

Beyond cytokine suppression, DHM may also reduce neuroinflammation by promoting Aβ clearance through microglial and inflammasome-associated mechanisms. Feng et al. (2018) showed that intraperitoneal DHM in APP/PS1 mice reduced microglial activation and NLRP3 inflammasome activity while promoting Aβ clearance through increased neprilysin (NEP) expression and a shift toward the anti-inflammatory M2 microglial phenotype [18]. Notably, DHM reduced Aβ deposition without altering APP or BACE1 expression, suggesting that its effect resulted from enhanced clearance rather than reduced Aβ production. Together, these findings link DHM-mediated Aβ clearance directly to suppression of NLRP3-associated microglial neuroinflammation.

DHM formulation strategies may further enhance these neuroprotective effects by improving its delivery and bioavailability. Li et al. (2025) developed two brain-targeting nanoparticle systems (TGN-DMY-CS/TPP-NPs and TGN-DMY-OVA/CMC-NPs) that both restored antioxidant enzyme levels (CAT and SOD), reduced TNF-α and IL-1β, preserved hippocampal neuronal integrity and synaptic density, and reversed mitochondrial dysfunction via the AMPK-mTOR pathway [21].

#### 2.1.2. Parkinson’s Disease

Parkinson’s disease (PD) is a neurodegenerative disorder driven by loss of dopaminergic neurons in the substantia nigra and accumulation of α-synuclein in Lewy bodies [49,50]. Effects of PD further extend to neurotransmitter systems and protein aggregates beyond the Lewy body itself [51]. Genetic and environmental factors are known to interact strongly in disease, but the precise cause of PD remains unknown, and no treatment currently exists that slows neurodegeneration [52]. Given its significant effects on the CNS, DHM has been investigated as a promising therapeutic candidate across interrelated aspects of PD: α-synuclein accumulation, dopaminergic neuron loss, and neuroinflammation.

Because α-synuclein accumulation contributes to glial-mediated neuroinflammation, DHM-mediated protein clearance may represent one mechanism linking autophagy to inflammatory regulation in PD. Wu et al. (2019) found that DHM activates chaperone-mediated autophagy (CMA) to degrade α-synuclein aggregates in BAC-α-syn-GFP transgenic mice, evidenced by increased LAMP-1/LAMP-2A levels and their colocalization with α-synuclein inclusions in vitro, alongside reduced astrogliosis and microgliosis in vivo [23]. This relationship between autophagy and neuroinflammation is further supported by Zhang et al. (2023), who showed in MPTP/probenecid-induced PD models that DHM stimulated autophagy while inhibiting pyroptosis and necroptosis, lowering α-synuclein levels alongside the inflammatory markers GFAP, Iba-1, TNF-α, and IL-6 [22]. Together, these findings identify autophagy as a recurring mechanism linking DHM-mediated protein clearance, inflammatory cell-death pathways, and glial activation in PD.

### 2.2. Neurovascular Disorders

#### 2.2.1. Stroke

Ischemic stroke (IS) is a cerebrovascular disorder characterized by a thrombotic or embolic event that restricts blood flow to the brain [53]. Following ischemic injury, microglia produce ROS, proinflammatory cytokines, and matrix metalloproteinases that disrupt BBB permeability and permit peripheral immune cell infiltration [54]. Resulting neuroinflammation is linked to increased risk of brain atrophy or stroke [55]. DHM is currently being investigated for its neuroprotective potential in IS across both in vitro and in vivo models, with two consistent findings emerging: convergence on NF-κB signaling in cell-based models, and broader anti-apoptotic, anti-pyroptotic effects in vivo.

In oxygen-glucose deprivation/re-oxygenation (OGD/R) models, DHM downregulates long noncoding RNAs implicated in neuroinflammation through distinct upstream routes that arrive at the same downstream target. Zeng et al. (2024) showed that DHM dose-dependently reduced SNHG10 overexpression, increasing cell viability and decreasing pro-inflammatory factors via the SNHG10/miR-665/RASSF5 axis, which converges on NF-κB [25]. Similarly, Xie et al. (2025) found that DHM reduced SNHG17 upregulation in OGD/R-treated BV2 microglia, inhibiting NF-κB activation via miR-452-3p/CXCR4 and decreasing NLRP3 inflammasome activation, pro-caspase-1 expression, cell death, and pro-inflammatory cytokines [26]. That these independent studies both show long noncoding RNA pathways that terminate at NF-κB provides preliminary evidence for mechanistic convergence on NF-κB.

This anti-inflammatory profile holds in in vivo IS models as well. Zhao et al. (2017) reported that DHM suppresses LPS-activated microglia, inhibits caspase-3 cleavage, and attenuates apoptosis and OGD-induced cell death while activating the ERK1/2-CREB pathway to upregulate neuroprotective genes like Bcl-2 [24]. Ding et al. (2023) linked a similar reduction in pyroptosis to fewer NLRP3-positive cells and lower IL-1β via activation of the Nrf2 pathway in Sprague-Dawley rats [27]. Similarly, Wasan et al. (2022) showed that intraperitoneal DHM improved motor function and reduced apoptosis after reperfusion injury, decreasing Bax and cleaved caspase-3 while also reducing reactive astrocytes and glial scar formation [28].

#### 2.2.2. Hemorrhage

Hemorrhagic strokes, encompassing subarachnoid hemorrhagic stroke (SAH) and intracerebral hemorrhagic stroke (ICH), cause cognitive and neurological decline with high mortality rates, driven by oxidative stress and progressive brain atrophy following the initial bleed [56]. Effective therapy therefore needs to target both the neuronal damage and proinflammatory response that the injury exacerbates. DHM has shown benefit in both models of SAH and ICH, though through distinct downstream mechanisms.

In SAH, Li et al. (2023) found that intraperitoneal DHM attenuated secondary injury processes, including oxidative stress and cytotoxicity, through activation of the Nrf2/Prx2 signaling pathway [35]. These protective effects were also confirmed in vitro in DHM-treated cortical neuronal cells. Similarly, in an ICH model, DHM reduced hematoma size, cell death, and behavioral deficits, but through a distinct molecular mechanism involving the system Xc^−^ pathway [36]. DHM suppressed expression of the ferroptosis-associated protein Lipocalin-2 (LCN2), thereby restoring cystine transport and GSH-GPX4 levels, inhibiting ferroptosis, and limiting oxidative tissue damage. Together, these findings suggest that DHM provides neuroprotection across hemorrhagic stroke models by regulating oxidative stress and cell death, although the specific molecular pathways involved may differ between SAH and ICH.

### 2.3. Psychiatric Disorders

#### 2.3.1. Depression

Neurodegenerative diseases, such as AD and PD, frequently present with comorbid depressive symptoms driven by underlying neuroinflammation. Major depressive disorder (MDD) is highly prevalent among these patients, with substantial impact on quality of life [57]. This motivates interest in therapeutics that can address neuroinflammation, a role DHM’s anti-inflammatory profile positions it to fill.

Several of DHM’s antidepressant effects converge on suppression of the NLRP3 inflammasome, reached through distinct upstream pathways depending on disease context. In LPS-treated mice, Wei et al. (2022) found that DHM reduced neuroinflammation and depressive-like behavior by suppressing TLR4/Akt/HIF-1α/NLRP3 signaling, alongside inhibition of NF-κB activation and pro-inflammatory cytokine production [29]. In a more specific comorbid context, a pre-clinical model of MPTP-induced PD with depression, Lü et al. (2026) traced DHM’s effect to the LCN2/NLRP3 axis, where DHM shifted astrocyte polarization from the neurotoxic A1 phenotype to the neuroprotective A2 phenotype, reversed neuronal loss, restored the synaptic markers BDNF, SYN1, and PSD-95, and alleviated ferroptosis [32].

DHM’s antidepressant activity extends beyond inflammasome regulation, converging instead on neurotrophic signaling in several models. In a corticosterone-induced depression model, Huang et al. (2022) showed that DHM suppressed advanced glycation end product (AGE) and receptor of AGE (RAGE) signaling, a corticosterone-upregulated pathway that drives proinflammatory cytokine production [30]. This inhibition reduced both cytokine levels and depressive-like behavior. These findings align with a broader BDNF-centered mechanism, as Ren et al. (2017) linked reduced brain-derived neurotrophic factor (BDNF) expression to MDD and demonstrated that DHM-mediated restoration of BDNF signaling may reduce associated neuroinflammation [31]. In both acute LPS-induced and chronic unpredictable mild stress (CUMS) models, intraperitoneal DHM improved depressive-like behavior by increasing ERK1/2-CREB and GSK-3β (Ser-9) phosphorylation and restoring BDNF expression, with similar effects observed in cultured hippocampal neurons. Together, these findings suggest that DHM’s antidepressant effects involve coordinated suppression of inflammatory signaling and restoration of neurotrophic pathways associated with BDNF.

#### 2.3.2. Social Isolation Induced Anxiety

Social isolation is closely linked to generalized anxiety, as prolonged isolation and loneliness erode a person’s support network and intensify comorbid depression and anxiety [58]. Recent work has connected anxiety disorders to both inflammatory signaling and cognitive decline, positioning DHM’s anti-neuroinflammatory profile as a candidate for alleviating social-isolation-induced anxiety specifically.

DHM’s protective effects in this model act on several converging cell populations. At the astrocytic level, DHM restores cognition and memory in socially isolated mice while decreasing GFAP, a marker of astrocytic reactivity [34]. A parallel effect occurs at the level of the GABAergic synapse itself. DHM restores the expression of gephyrin, a protein essential for GABAergic neurotransmission that social isolation suppresses, alongside reduced hippocampal microglial activity and decreased corticosterone-driven NF-κB signaling [33]. Together, these findings indicate DHM can alleviate social isolation-induced anxiety-like behavior by targeting both microglial neuroinflammation and GABAergic synaptic integrity.

### 2.4. Neuropathic Pain

Neuropathic pain commonly arises as a consequence of various diseases or physical injury, which can result in recurrent noxious stimulation, inflammation-induced pain, or persistent pain even after the resolution of inflammatory responses [59]. Among these, diabetes is a well-recognized cause of neuropathic pain, often referred to as diabetic neuropathy. Recent studies have demonstrated that DHM possesses potential for mitigating neuropathic pain across both diabetic and non-diabetic models, with converging evidence pointing to its capacity to suppress neuroinflammation.

In diabetic neuropathic pain models, P2X7 emerges as a central node. Ge et al. (2020) showed that DHM reduced astrocytic damage and P2X7 receptor signaling, suggesting a role for P2X7-mediated astrocytic suppression in DHM’s effects [38]. A similar pattern was reported in comorbid diabetic neuropathic pain (DNP) and depression models. The group also found that DHM improved thermal withdrawal latency and mechanical withdrawal threshold while normalizing DNP-associated BDNF and TrkB changes in the spinal cord and DRG, alongside reduced IL-1β and TNF-α [39]. Guan et al. (2019) reported that P2X7 knockdown reproduced DHM’s effects: both DHM and P2X7 knockdown produced comparable reversal of pain behaviors, reduced P2X7 expression, lowered serum TNF-α and IL-1β, and reversed elevated p-ERK1/2 in the DRG and spinal cord, consistent with P2X7-mediated ERK inhibition as a proposed downstream mechanism [41].

In non-diabetic neuropathic pain models, DHM’s action shifted to microglial polarization. Zhang et al. (2020) reported microglial ALDH2-mediated M1 to M2 polarization as the proposed mechanism: in LPS-stimulated BV-2 microglia, DHM reduced M1 markers (iNOS and CD86) while elevating M2 markers (Arg1 and CD206) and ALDH2 expression, an effect reproduced by the ALDH2 agonist ALDA-1 [37]. Similarly, in a spared nerve injury (SNI) model, DHM restored M2 polarization markers and ALDH2 levels alongside increased paw withdrawal threshold [37]. Li et al. (2026) added a further layer using network pharmacology, identifying VEGFA as a high-ranked intersecting target between DHM, neuropathic pain, and depression [40]. DHM restored SNI-reduced hippocampal VEGFA/VEGFR2 expression alongside reduced IL-1β and TNF-α, suggesting VEGFA/VEGFR2 signaling as a possible additional route through which DHM may attenuate central neuroinflammation and pain.

### 2.5. Emerging Disease Contexts

#### 2.5.1. Spinal Cord Injury

Spinal cord injury (SCI) results from mechanical compression or disruption of the cord, impairing motor, sensory, and autonomic function. Injury triggers activation of microglia, astrocytes, and leukocytes, releasing cytokines (TNF-α, IL-1β) and chemokines (CCL2, CXCL10) that initially control damage and promote repair [60]. However, this response can become chronic, as neutrophils and macrophages release proteases and oxidative metabolites that break down the BBB [61], increasing permeability and permitting further immune infiltration in a self-perpetuating cycle. Recent work indicates DHM interrupts this cycle by suppressing the NLRP3/Caspase-1 inflammasome, reaching that target through both a proposed direct, local route within the spinal cord and an indirect, systemic route via the gut.

Locally, DHM-associated inflammasome suppression has been proposed to involve inhibition of STING (stimulator of interferon genes), which may block the PI3K/AKT pathway to promote autophagy and reduce microglial pyroptosis and oxidative stress [42]. In mice with surgically induced spinal cord compression, DHM improved motor function and reduced tissue damage by decreasing pyroptosis-related proteins (IL-18, NLRP3, ASC, GSDMD, Caspase-1) and increasing oxidative stress defense proteins SOD1 and HO-1. Because DHM was given systemically, these tissue-level changes are associations and do not distinguish direct from indirect effects. In vitro, in BV-2 microglia, DHM promoted autophagy (↑ Beclin1, VPS34, CTSD, LC3II; ↓ p62) while suppressing oxidative stress and pyroptosis, an effect reversed by the autophagy inhibitor 3-MA, suggesting autophagy as the operative mechanism.

Systemically, DHM reaches the same inflammasome endpoint by acting on the gut microbiome [43]. In SCI rats, DHM improved forelimb function recovery and preserved spinal cord tissue structure while reversing gut microbiota disruption, increasing butyrate-producing genera and fecal butyric acid, and restoring intestinal barrier integrity (↑ Occludin, ZO-1). This corresponded with reduced circulating lipopolysaccharide (LPS), suppressed TLR4/MyD88/NF-κB signaling, and reduced NLRP3/Caspase-1 inflammasome activity and inflammatory cytokines (IL-1β, TNF-α) in spinal cord tissue. This route may not require BBB penetration to produce its effect, suggesting that DHM’s peripheral, gut-mediated action and its more directly CNS-targeted mechanisms (e.g., in AD models) may represent at least partially distinct pathways, although the studies reviewed here did not test whether CNS exposure was required and do not rule out concurrent central exposure occurring alongside the peripheral mechanism.

#### 2.5.2. Epilepsy

Epilepsy is characterized by recurrent seizures and is frequently accompanied by comorbid cognitive impairment, with neuroinflammation implicated in both seizure recurrence and the associated hippocampal damage [62]. In a pilocarpine-induced status epilepticus rat model, Li et al. (2025) found that DHM dose-dependently reduced the frequency and duration of spontaneous recurrent seizures and improved long-term potentiation and cognitive deficits following status epilepticus [44]. DHM increased expression of synaptic proteins and protected hippocampal pyramidal neurons from seizure-induced damage, with this neuroprotective effect proposed to involve inhibition of NLRP3-associated pyroptosis. These findings position epilepsy as an additional disease context in which DHM’s core anti-inflammatory mechanism, NLRP3 inflammasome suppression, extends to seizure-related neuroinflammation and its cognitive sequelae.

## 3. Discussion

Across the disease contexts reviewed, DHM treatment was repeatedly associated with changes in a relatively small set of recurring targets, with the most common being suppression of the NF-κB pathway [19,24,25,26,29,33,43], inhibition of the NLRP3 inflammasome [18,26,27,29,32,42,43,44], and modulation of autophagy [22,23,42]. This pattern was not uniform across all disease contexts. Studies of hemorrhagic stroke, neuropathic pain, and anxiety focused predominately on other pathways, including Nrf2-linked oxidative stress and ferroptosis control in hemorrhagic stroke [35,36], P2X7/ERK signaling and ALDH2-mediated microglial polarization pathways in neuropathic pain [37,41], and GABAergic signaling and astrocyte reactivity in anxiety, despite measurable NF-κB suppression [33,34]. Depression was classified as convergent because NLRP3 suppression was a principal finding, but it also involved neurotrophic signaling not encompassed by the NF-κB/NLRP3/autophagy model [29,30,31,32]. Whether this reflects genuine mechanistic divergence or simply reflects which pathways each study happened to measure is unclear from the current literature. In broader mechanistic literature, NF-κB and NLRP3 are functionally linked; NF-κB activation drives transcription of NLRP3 components, and, in turn, NLRP3 inflammasome activity amplifies NF-κB signaling [63]. Autophagy serves as a negative regulator of both pathways by removing damaged organelles and inflammasome components that would continue the cycle of inflammation [64]. The repeated association of DHM with these three processes across distinct disease contexts suggests a potentially shared inflammatory framework.

**Figure 1:**
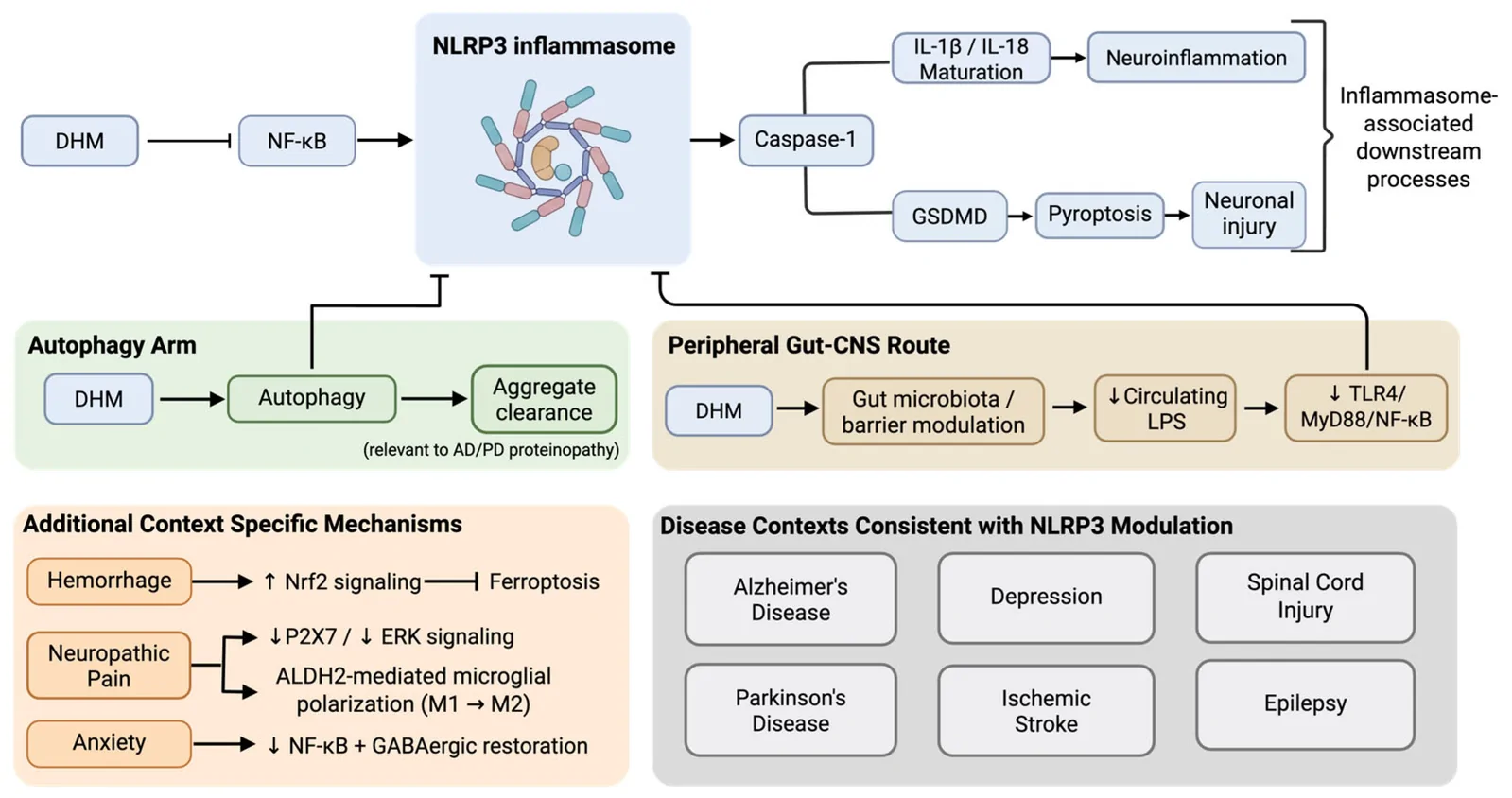
Proposed integration of NLRP3-centered and context-specific mechanisms of DHM in CNS disease and injury. Dihydromyricetin (DHM) has been reported to modulate the NLRP3 inflammasome through three upstream routes: suppression of NF-κB signaling, promotion of autophagy, and modulation of a peripheral gut–CNS axis involving reduced circulating lipopolysaccharide (LPS) and TLR4/MyD88/NF-κB signaling. These routes may converge on reduced NLRP3 inflammasome activity and associated downstream processes, including caspase-1-dependent IL-1β/IL-18 maturation and neuroinflammation, as well as GSDMD-mediated pyroptosis and neuronal injury. The autophagy arm additionally promotes aggregate clearance relevant to Alzheimer’s disease (AD) and Parkinson’s disease (PD) proteinopathy. The (**lower-left panel**) presents additional mechanisms reported in specific disease contexts, including Nrf2-associated inhibition of ferroptosis in hemorrhage, modulation of P2X7/ERK signaling and ALDH2-mediated microglial polarization in neuropathic pain, and suppression of NF-κB signaling with restoration of GABAergic/BDNF signaling in anxiety. The (**lower-right panel**) identifies CNS disease and injury contexts in which NLRP3 or other components of the proposed NF-κB/NLRP3/autophagy circuit have been reported; direct NLRP3 evidence for each context is indicated in Table 1. Arrowheads indicate activation or downstream progression, whereas blunt-ended connectors indicate inhibition or suppression. Individual signaling relationships depicted in the figure were experimentally reported in the cited primary studies; however, their organization into a shared, convergent NLRP3-centered network represents the authors’ proposed integration of findings across independent studies and has not been demonstrated in its entirety within a single experimental system.

Importantly, this model is intended to be hypothesis-generating and does not represent an experimentally established unified pathway; its proposed components and interactions require validation through targeted mechanistic studies. Figure 1 presents a proposed model of this mechanistic convergence, integrating the NF-κB/NLRP3/autophagy circuit with the peripheral gut–CNS route and predominant alternative mechanisms identified across the reviewed studies. Table 2 classifies the nine disease contexts by their predominant reported mechanism. Contexts were classified as convergent when a core-circuit component (NF-κB/NLRP3/autophagy) was the principal reported mediator and as predominantly alternative when the principal reported mediator lay outside the circuit, even where a core component also changed. On this basis, six contexts (AD, PD, ischemic stroke, depression, SCI, and epilepsy) were convergent while three (hemorrhage, neuropathic pain, and anxiety) were predominately alternative; depression and anxiety were boundary cases. This hypothesis-generating convergence framework distinguishes the present synthesis from prior pharmacological reviews of DHM (e.g., Zhang et al., 2025), which catalog the compound’s anti-inflammatory, antioxidant, and anti-apoptotic effects as parallel findings across disease states without evaluating whether these effects converge on a shared mechanism [16]. This review further incorporates literature published subsequent to Zhang et al. (2025) [16], including recent studies examining DHM’s role in spinal cord injury, epilepsy, and comorbid depression models [32,42,43,44].

DHM’s capacity to cross the BBB allows it to produce neuroprotective effects through both direct central and indirect peripheral mechanisms. In rats, oral administration of DHM resulted in rapid brain distribution, with detectable concentrations enduring for up to 12 h [13]. Similarly, in mice, DHM and two O-methylated metabolites were detected in brain tissue 15 min after administration [14]. These findings establish the potential for direct CNS activity; however, DHM may also influence neurological outcomes indirectly through effects on peripheral processes that contribute to CNS injury. The mechanistic duality of DHM is most clearly illustrated by the two SCI studies discussed in this review: Liu et al. (2025) found that DHM administration was associated with reduced STING expression and pyroptosis-related proteins in SCI mice, with in vitro microglial experiments supporting a STING-PI3K/AKT-autophagy pathway, while Yang et al. (2026) reported a peripheral route, in which DHM’s remodeling of the gut microbiome reduced circulating LPS, suppressed TLR4/NF-κB signaling, and lessened spinal cord inflammasome activity [42,43]. That these two studies, examining the same compound in the same injury model, arrived at independent explanations for the same therapeutic outcome raises the possibility that DHM’s central and peripheral actions are not mutually exclusive, though neither study excludes potential contribution from the other route. While DHM’s suppression of TLR4 via gut microbiota modulation may not require CNS penetration to produce its effect, its direct inhibition of microglial TLR4/MD2 signaling in AD models [17] is consistent with central action at the same molecular target through a different route. This dual capacity may allow DHM to address neuroinflammation at the site of injury while also weakening upstream peripheral inflammatory inputs that would otherwise sustain CNS inflammation. Whether these two routes of action are additive, synergistic, or context-dependent remains an open question for future studies. This central-peripheral duality, together with the inclusion of spinal cord injury and gut-microbiome-mediated signaling more broadly, has not been addressed in prior DHM reviews and represents a mechanistic dimension unique to the present synthesis.

Beyond this central-peripheral duality, DHM’s established activity as a positive allosteric modulator of the GABA-A receptor raises the possibility of a third, mechanistically distinct route contributing to its anti-inflammatory effects [65]. Studies have shown that astrocytes and microglia express GABA-A receptors, suggesting that glia cell function may be directly influenced by GABAergic signaling [66]. GABA-A receptor activation in astrocytes and microglia has been associated with suppression of inflammatory responses and NF-κB signaling [66]. Given this evidence, DHM may interact via GABA-A receptors expressed on neuroimmune cells, providing a potential mechanism through which it could modulate microglial and astrocytic inflammatory activation. This proposed interaction may contribute to the observed anti-inflammatory effects of DHM. However, further mechanistic studies should be done to determine whether DHM directly engages with GABA-A receptors on microglia or astrocytes to regulate neuroinflammation. Additionally, given that GABAergic signaling plays a role in regulating HPA-axis activity and corticosterone release, DHM may indirectly influence in corticosterone-associated inflammatory activation through its modulation of GABAergic signaling [33]. This proposes a potential link in the reduction of corticosterone-driven NF-κB and microglial activation. Together, these findings suggest that DHM’s neuroinflammatory effects may arise through multiple interacting central and peripheral mechanisms rather than a single molecular pathway. However, the extent to which these proposed mechanisms contribute to DHM’s effects in vivo remains to be established.

While the studies reviewed demonstrate DHM’s neuroprotective effects across a range of CNS disease contexts, several consistent limitations weaken the strength of these conclusions. Importantly, the current literature is almost entirely preclinical: the majority of studies rely on acute animal injury models or in vitro cell-line systems, with no human clinical data on DHM’s neuroinflammatory effects. These model systems may not fully capture the complexity or chronicity of human CNS disease. The translational relevance of the DHM concentrations used in the available in vitro studies remains uncertain because corresponding total and unbound plasma and brain exposures have not been consistently characterized [37,38]. This raises the question of whether the observed effects would be reproducible at clinically relevant exposure levels. Dosing, route of administration, treatment duration, and outcome measures also vary considerably across studies, making cross-study comparison difficult and complicating efforts to identify an optimal therapeutic method. Additional limitations include the predominance of male animals, limited evaluation of sex-dependent responses, and the use of immortalized BV2 microglia and other cell-line models in several studies, which may not fully reproduce the biology of primary CNS cells. Small sample sizes and limited replication across independent laboratories further restrict the strength and generalizability of the evidence. The evidence base may also be subject to publication bias, as studies reporting beneficial effects of DHM may be more likely to be published than those reporting null or negative findings. Moreover, apparent mechanistic convergence or divergence may partly reflect selective pathway measurement, as frequently examined pathways may appear disproportionately prominent while unmeasured pathways cannot be assumed to be absent.

A recurring challenge across the reviewed studies is DHM’s unfavorable pharmacokinetic profile, rooted in its underlying chemical structure. As a flavonoid, DHM is defined by a three-ring heterocyclic structure, substituted with six hydroxyl groups. These groups engage in intermolecular hydrogen bonding, contributing to a stable crystal lattice that can reduce the accessibility of its phenolic hydroxyls for hydration, thereby limiting aqueous dissolution and contributing to DHM’s poor aqueous solubility (0.2 mg/mL at 25 °C) [67]. This, in turn, decreases the intestinal absorption of the compound and its oral dosing capabilities. DHM’s insolubility contributes to its unfavorable pharmacokinetic profile, characterized by a low C_max_ (21.63 ± 3.62 ng/mL, approximately 0.07 μM) and short half-life (t_1_/_2_: 3.70 ± 0.99 h) following a 20 mg/kg oral dose prepared in an unnamed oral suspension vehicle in male Sprague-Dawley rats [68]. Higher oral exposure has been reported in mice given 50 mg/kg DHM dissolved in saline with 10% DMSO, with sex-dependent serum C_max_ values of approximately 0.62 µM (female) and 1.9 µM (male) [14], although differences in species, dose and formulation limit direct comparison. For context, several of the in vitro studies reviewed here evaluated DHM at micromolar concentrations, frequently ranging from 1 to 100 µM (Table S1) and substantially exceed these reported total plasma C_max_ values. However, direct comparison is limited because total plasma concentrations do not reflect unbound drug exposure or brain concentrations, which were not consistently reported across studies. DHM is also easily susceptible to decomposition from temperature changes, pH buffers, metal ions (Fe^3+^, Al^3+^, Cu^2+^), and pancreatic enzymes [69]. Therefore, CNS exposure after oral administration remains constrained by poor systemic bioavailability. Many in vivo CNS studies bypass this bioavailability problem by using intraperitoneal injection rather than oral administration, which, while effective experimentally, is not a clinically viable route for chronic CNS conditions.

Novel delivery systems represent promising solutions to this gap. For example, co-crystallization with triethanolamine disrupts DHM’s hydrogen-bonding network, resulting in significantly improved water solubility, absorption, and distribution [70]. Other delivery systems, such as solid dispersions, nanocapsules [71], microemulsions [72], and cyclodextrin inclusion complexes [73], have also proven effective in enhancing solubility and enabling controlled release. However, these formulations have mainly been evaluated in acute disease models, and their performance in chronic neuroinflammatory settings remains largely unexplored. Future research should therefore test these delivery systems under conditions representative of long-term clinical use.

Safety represents another important consideration for the clinical potential of DHM. Available evidence suggests favorable tolerability of DHM, although safety data remain limited. Purified DHM has demonstrated low acute toxicity in mice, with an oral LD_50_ > 5 g/kg and no significant acute toxicity or adverse effects reported at doses ranging from 150 mg/kg to 1.5 g/kg [74]. Meanwhile, longer term evidence is more limited and includes botanical preparations containing DHM. For example, rats administered *Ampelopsis grossedentata* for 12 weeks showed no significant changes in body weight or blood biochemistry, and no delayed toxicity after drug cessation, although these findings cannot be attributed to purified DHM [75]. Small human studies administering DHM for several months have similarly reported no treatment-related adverse events, but were not designed to establish long term safety [76]. Potential drug interactions also remain insufficiently characterized, with in vitro evidence of CYP enzyme inhibition but limited in vivo interaction data [77,78]. Therefore, the safety of chronic DHM administration and potential drug–drug interactions merits further investigation.

More broadly, the field would benefit from greater use of longitudinal study designs in chronic disease models, which would better reflect the disease trajectories of conditions like AD, PD, and depression rather than the acute or induced models widely used in the literature. Additionally, human clinical trials are an absent but necessary step in establishing the safety, pharmacokinetics, and therapeutic potential of DHM, namely whether DHM’s preclinical neuroinflammatory potential translates to patients.

## 4. Materials and Methods

A comprehensive literature search was conducted to identify studies investigating DHM in CNS-related disorders and cellular models. Initial searches used PubMed and Google Scholar and focused on studies published up to June 2026. Primary search terms included combinations of “dihydromyricetin” and “neuroinflammation”, “Alzheimer’s Disease”, “Parkinson’s Disease”, “stroke”, “hemorrhage”, “depression”, “anxiety”, “neuropathic pain”, “epilepsy”, “spinal cord injury”, “central nervous system”, and “microglia”. Studies were included if the investigated therapeutic or biological effect of DHM was primarily mediated through modulation of neuroinflammatory processes, including direct regulation of pro- or anti-inflammatory signaling cascades, modulation of inflammatory cytokines or chemokines, regulation of microglial or astrocytic inflammatory activation states, and modulation of pattern recognition receptors involved in neuroinflammation. Studies were excluded if the predominant mechanism of DHM action was non-inflammatory, including metabolic pathways and neuronal mechanisms where inflammation was not demonstrated as a downstream consequence or parallel mechanism. Non-inflammatory mechanisms were retained when the study also demonstrated an inflammatory consequence or when the mechanism directly intersected with an established neuroinflammatory process. Study selection and screening were performed collaboratively among the author team; borderline studies were discussed among the authors and resolved by consensus based on the inclusion criteria above. From each included study, data were extracted on disease state or model, specific inflammatory pathways regulated, inflammatory mediators measured, cell types or animal models involved, and key findings related to neuroinflammation. Given the narrative nature of this review, no formal quality assessment was applied.

## 5. Conclusions

Collectively, the available preclinical evidence supports the continued investigation of DHM as a potential therapeutic candidate for CNS conditions involving neuroinflammation. This potential is supported by its engagement of multiple conserved inflammatory nodes across diverse disease models and its mechanistic reach across both central and peripheral inflammatory routes [12]. However, whether these effects translate to clinically meaningful benefit remains unknown. Establishing the translational potential of DHM will require pharmacokinetic-based dosing strategies, longitudinal chronic disease models, and ultimately clinical validation. DHM therefore represents a promising natural product for further investigation, but additional pharmacokinetic, mechanistic, and human studies are needed before its therapeutic relevance to CNS neuroinflammatory disease can be established.

## Figures and Tables

**Table 1:** Summary of DHM-associated molecular and cellular changes, associated pathways, and mechanistic intervention evidence across the CNS disease and injury contexts reviewed.

Disease	Experimental Model(s)	DHM-Associated Molecular and Cellular Changes ^1^	Associated Pathway(s)/Target(s)	Mechanistic Intervention ^2^	Citation
Alzheimer’s Disease	APP/PS1 Double Transgenic AD Mice; MD2 knockdown BV2 cells	↓ TLR4 and MD2 expression; ↓ IL-1β, IL-6, and TNF-α; DHM effects abolished by MD2 knockdown	TLR4/MD2	Interventional (in vitro only): MD2 knockdown abolished DHM’s anti-inflammatory effects in BV2 cells	[17]
	APP/PS1 Double Transgenic AD Mice; BV2 Cells	↓ CD68 and NLRP3 inflammasome activation; ↑ Aβ clearance via NEP; M2 microglial polarization; ↓ IL-1β and TNF-α	NLRP3	Associative: no pathway-specific intervention reported	[18]
	Sprague-Dawley rats (intracerebral Aβ1–42 injection)	↓ IL-1β, IL-6, and TNF-α; restored p-AMPK/AMPK and SIRT1; ↓ NF-κB p65; ↑ Bcl-2 and ↓ Bax	AMPK/SIRT1	Associative: no pathway-specific intervention reported	[19]
	Sprague-Dawley rats (ICV Aβ1–42 infusion)	↓ IL-1β, IL-6, and TNF-α	Not specified (cytokine readout only)	Associative: no pathway-specific intervention reported	[20]
	Obesity-related AD mice; TGN-DMY-CS/TPP-NPs and TGN-DMY-OVA/CMC-NPs	↓ brain neuroinflammation and oxidative stress; ↓ TNF-α and IL-1β; ↑ CAT and SOD; improved mitochondrial function and synaptic integrity	AMPK-mTOR	Associative: no pathway-specific intervention reported	[21]
Parkinson’s Disease	MPTP/probenecid-treated C57BL/6 mice; BV-2 cells	↓ GFAP and Iba-1; ↓ TNF-α and IL-6; ↓ NLRP3; ↑ autophagy; ↓ pyroptosis and necroptosis	Autophagy-mediated inhibition of pyroptosis/necroptosis	Interventional (in vitro only): 3-MA blunted DHM’s anti-inflammatory effect	[22]
	BAC-α-syn-GFP transgenic mice; human H4 neuroglioma cells	↑ CMA-mediated α-synuclein degradation; ↑ LAMP-1 and LAMP-2A; ↓ astrogliosis and microgliosis in vivo	CMA/α-synuclein degradation	Associative: no pathway-specific intervention reported	[23]
Ischemic Stroke	Male ICR mice (MCAO/reperfusion); LPS-stimulated BV2 microglia; HT22 neuronal cells (OGD/R)	↓ microglial activation; ↓ NF-κB p65, iNOS, COX-2, TNF-α, IL-1β, and IL-6; ↓ neuronal apoptosis and caspase-3 cleavage; ↑ ERK1/2–CREB–Bcl-2 signaling	Microglial NF-κB; neuronal ERK1/2–CREB–Bcl-2	Interventional (in vitro only): U0126 abolished DHM-mediated neuroprotection; microglial NF-κB remained associative	[24]
	SH-SY5Y cells (OGD/R)	↓ lncRNA SNHG10; ↑ cell viability; ↓ pro-inflammatory mediators and NF-κB activation	SNHG10/miR-665/RASSF5/NF-κB	Interventional: SNHG10 overexpression partially reversed DHM protection; miR-665 mimic and RASSF5 knockdown rescued it	[25]
	BV2 microglia (OGD/R); HEK293 cells	↓ SNHG17 and CXCR4; ↓ NF-κB and NLRP3 activation; ↓ pro-caspase-1, oxidative stress, and pro-inflammatory cytokines	SNHG17/miR-452-3p/CXCR4/NF-κB	Interventional: SNHG17 overexpression reversed DHM protection; miR-452-3p mimic enhanced it, and CXCR4 knockdown restored it	[26]
	Male Sprague-Dawley rats	↑ Nrf2 activation; ↓ NLRP3 inflammasome activation and pyroptosis; ↓ NLRP3-positive cells and IL-1β	Nrf2/NLRP3	Associative: no pathway-specific intervention reported	[27]
	Male Wistar rats (ischemia/reperfusion)	↓ reactive astrocytes and glial scar formation; ↓ Bax and cleaved caspase-3; ↑ Bcl-2; improved motor function	Intrinsic apoptotic pathway	Associative: no pathway-specific intervention reported	[28]
Depression	Male C57BL/6 mice (LPS-induced depression); LPS-stimulated BV2 microglia	↓ CD11b, TNF-α, IL-6, IL-1β, COX-2, and iNOS; ↓ TLR4/CD14, p-Akt, HIF-1α, and NF-κB activation; ↓ NLRP3, ASC, and caspase-1; improved sickness and depressive-like behaviors	TLR4/NF-κB/Akt/HIF-1α/NLRP3; NF-κB	Associative: no pathway-specific intervention reported	[29]
	Male ICR mice (corticosterone-induced depression)	↓ AGE and RAGE expression; ↓ hippocampal IL-1β, IL-6, and TNF-α; improved depressive-like behavior	AGE/RAGE signaling	Associative: no pathway-specific intervention reported	[30]
	Male C57BL/6 mice (acute LPS-induced and CUMS depression); primary hippocampal neurons	↑ BDNF expression; ↑ ERK1/2-CREB and GSK-3β (Ser-9) phosphorylation; ↓ hippocampal TNF-α and IL-6; improved depressive-like behavior	ERK1/2-CREB-BDNF/GSK-3β	Interventional (in vitro only): U0126 and LY294002 inhibited DHM-stimulated BDNF expression	[31]
	MPTP-induced PD with depression mouse model; C8-D1A astrocytes (MPP^+^); SN4741 dopaminergic neurons (astrocyte-conditioned medium)	↓ LCN2/NLRP3 signaling; A1 → A2 astrocyte polarization (↓ C3; ↑ S100A10); ↓ ferroptosis (↓ ACSL4, TFRC; ↑ GPX4, SLC7A11); ↑ BDNF, SYN1, and PSD-95; ↓ neuronal loss	LCN2/NLRP3/astrocyte polarization	Interventional (in vitro only): NLRP3 inhibitor MCC950 reproduced DHM’s astrocyte-polarization and neuronal-ferroptosis changes	[32]
Social Isolation Induced Anxiety	Male C57BL/6 mice (4–6 weeks social isolation); ex vivo hippocampal assays	↓ microglial activation; ↓ NF-κB signaling and pro-inflammatory cytokines; ↓ serum corticosterone; ↑ gephyrin expression/GABAergic function; improved anxiety-like behavior	NF-κB; GABAergic signaling; HPA axis	Associative: no pathway-specific intervention reported	[33]
	Male C57BL/6 mice (4 weeks social isolation)	↓ GFAP/astrocyte reactivity; restored astrocyte morphology and complexity; ↑ astrocyte-mediated synaptic support and plasticity; improved memory and cognition	Astrocyte reactivity	Associative: no pathway-specific intervention reported	[34]
Subarachnoid hemorrhage (SAH)	Adult Sprague-Dawley rats (prechiasmatic cistern blood injection); primary cortical neurons (oxyhemoglobin-induced injury)	↑ Nrf2 and Prx2; ↓ ASK1/p38 MAPK signaling and ROS; improved mitochondrial function; ↓ neuronal apoptosis (↓ Bax, caspase-3; ↑ Bcl-2); improved neurological outcomes	Nrf2/Prx2/ASK1/p38	Interventional: Prx2 siRNA (in vivo only) and Nrf2 inhibitor ML385 (in vivo and in vitro) attenuated DHM’s neuroprotection	[35]
Intracerebral hemorrhage (ICH)	Male C57BL/6 mice (collagenase-induced ICH)	↓ LCN2, ROS, COX-2, and p-ERK; restored system Xc^−^-mediated cystine transport and GSH/GPX4; ↓ ferroptosis, neuronal apoptosis, and hematoma volume	LCN2/system Xc^−^/GSH-GPX4	Interventional: LCN2 overexpression (adenovirus) reversed DHM’s reductions in COX-2, p-ERK, iron deposition and abnormal mitochondria	[36]
Neuropathic Pain	Male CD1 mice (SNI model); BV2 cells	M1 → M2 microglial polarization (↓ iNOS, CD86; ↑ Arg1, CD206); ↑ ALDH2 expression; ↑ paw withdrawal threshold	ALDH2-mediated microglial polarization	Interventional: ALDH2 agonist ALDA-1 reproduced DHM’s M1-to-M2 shift	[37]
	Primary hippocampal astrocytes (high glucose + substance P + corticosterone)	↓ P2X7 receptor expression; ↓ TNF-α and IL-1β; ↓ ERK1/2 phosphorylation; ↓ astrocyte apoptosis	P2X7/Ca^2+^-ERK1/2	Interventional: P2X7 shRNA reproduced DHM’s reduction of TNF-α, IL-1β, Ca^2+^ and p-ERK1/2	[38]
	Male Sprague-Dawley rats (diabetic neuropathic pain + chronic unpredictable stress)	↓ IL-1β and TNF-α in hippocampus, spinal cord, and DRG; ↓ BDNF/TrkB overexpression; improved thermal and mechanical pain thresholds	BDNF/TrkB	Associative: no pathway-specific intervention reported	[39]
	Male C57BL/6J mice (SNI; comorbid neuropathic pain and depression)	↑ hippocampal VEGFA/VEGFR2 expression; ↓ hippocampal IL-1β and TNF-α; ↑ paw withdrawal threshold	VEGFA/VEGFR2	Associative: no pathway-specific intervention reported	[40]
	Male Sprague-Dawley rats (comorbid diabetic neuropathic pain and depression); DRG and spinal cord	DHM and P2X7 knockdown produced comparable effects; ↓ P2X7 mRNA/protein; ↓ serum TNF-α and IL-1β; ↓ p-ERK1/2; improved MWT and TWL	P2X7/ERK1/2	Interventional (in vivo): P2X7 shRNA reproduced DHM’s behavioral relief and its reduction of p-ERK1/2, TNF-α and IL-1β	[41]
Spinal Cord Injury	C57BL/6 mice (spinal cord compression); BV-2 microglia (H_2_O_2_-induced injury)	↓ microglial pyroptosis (↓ NLRP3, ASC, GSDMD, caspase-1, IL-18); ↑ autophagy (↑ Beclin1, VPS34, CTSD, LC3II; ↓ p62); ↑ SOD1 and HO-1; ↓ oxidative stress; improved motor function	STING/PI3K/AKT/autophagy	Interventional (in vitro only): 3-MA counteracted DHM; STING siRNA/overexpression and PI3K activator 740 Y-P tested the STING-PI3K/AKT link	[42]
	Sprague-Dawley rats (C5 unilateral clip-compression SCI); antibiotic-depleted rats (FMT)	↑ butyrate-producing gut microbiota and fecal butyrate; ↑ intestinal barrier integrity (↑ Occludin, ZO-1; ↓ serum FITC-dextran); ↓ circulating LPS; ↓ TLR4/MyD88/NF-κB signaling and NLRP3/caspase-1 inflammasome activity; DHM-donor FMT transferred the reduced TLR4/NF-κB and NLRP3 readouts	Gut microbiota/LPS/TLR4/MyD88/NF-κB/NLRP3	Interventional: DHM-donor FMT transferred reduced NF-κB/NLRP3 signaling	[43]
Epilepsy	Male Sprague-Dawley rats (Pilocarpine-induced status epilepticus)	↓ SRS frequency/duration; improved LTP and cognitive deficits; ↑ synaptic protein expression; protected hippocampal pyramidal neurons; ↓ NLRP3-associated pyroptosis	NLRP3/Pyroptosis	Associative: no pathway-specific intervention reported	[44]

^1^ Entries summarize molecular, cellular, and behavioral changes reported following DHM treatment and do not, by themselves, establish mechanistic causality. ^2^ The final column identifies the pathway-specific intervention reported in each study (inhibitor, agonist, knockdown or overexpression, rescue, or cell or microbiota transfer) and its setting (in vitro or in vivo). Entries marked “Associative” reported parallel biomarker or behavioral changes only. ↑ and ↓ indicate increases and decreases, respectively, in the indicated molecular or cellular parameters following DHM treatment relative to the corresponding disease-model controls.

**Table 2:** Classification of disease contexts by predominant reported mechanism relative to the proposed NF-κB/NLRP3/autophagy circuit.

Disease State	Predominant DHM-Associated Mechanism	Molecular Nodes	Relationship to Proposed Core Circuit ^1^	Citation(s)
Alzheimer’s disease	Suppression of microglial inflammatory signaling and NLRP3 activation, with enhanced Aβ clearance	TLR4/MD2; NF-κB; NLRP3	Convergent	[17,18]
Parkinson’s disease	Autophagy-mediated α-synuclein clearance and suppression of inflammatory cell-death pathways	CMA/autophagy; pyroptosis/necroptosis	Convergent	[22,23]
Ischemic stroke	NF-κB suppression through distinct lncRNA pathways, with downstream NLRP3 inhibition	SNHG10; SNHG17; NF-κB; NLRP3	Convergent	[25,26]
Hemorrhage (SAH/ICH)	Regulation of oxidative stress, ferroptosis, and associated neuronal injury	Nrf2/Prx2; LCN2/System Xc^−^/GSH-GPX4	Predominantly alternative	[35,36]
Depression	NLRP3 suppression alongside restoration of neurotrophic signaling	NLRP3; LCN2; BDNF-associated signaling	Convergent ^2^	[29,32]
Anxiety	NF-κB suppression and restoration of GABAergic signaling, alongside reduced astrocyte reactivity	NF-κB; gephyrin; GFAP/astrocyte reactivity	Predominantly alternative ^3^	[33,34]
Neuropathic pain	P2X7/ERK regulation and ALDH2-mediated microglial polarization	P2X7/ERK; ALDH2	Predominantly alternative	[37,41]
Spinal cord injury	NLRP3/Caspase-1 suppression through local autophagic and peripheral gut-inflammatory mechanisms	STING/PI3K/AKT/autophagy; TLR4/MyD88/NF-κB/NLRP3	Convergent	[42,43]
Epilepsy	NLRP3-mediated pyroptosis inhibition	NLRP3 → Pyroptosis	Convergent	[44]

^1^ “Convergent” indicates that a core-circuit component (NF-κB, NLRP3, or autophagy) was the principal mediator; it does not imply that the complete circuit was experimentally demonstrated within an individual disease model. “Predominantly alternative” indicates that the principal reported mediator lay outside the circuit, even where a core component also changed. ^2^ Depression shows NLRP3-associated changes (via TLR4- and LCN2-linked routes) but also involves a substantial BDNF/neurotrophic signaling component not captured by the core NF-κB/NLRP3/autophagy circuit. ^3^ Anxiety shows measurable NF-κB suppression, but its predominant reported mechanism was restoration of GABAergic transmission and reduced astrocyte reactivity. Whether these operate independently of the core circuit was not tested.

## Data Availability

No new data were created or analyzed in this study. Data sharing is not applicable to this article.

## Supplementary Materials

The following supporting information can be downloaded at https://www.mdpi.com/article/10.3390/ijms27209080/s1.

## Abbreviations

The following abbreviations are used in this manuscript:

Aβ	Amyloid beta
AD	Alzheimer’s disease
AGE	Advanced glycation end product
Akt	Protein kinase B
ALDH2	Aldehyde dehydrogenase 2
ASC	Apoptosis-associated speck-like protein containing a CARD
BBB	Blood–brain barrier
BDNF	Brain-derived neurotrophic factor
CMA	Chaperone-mediated autophagy
COX-2	Cyclooxygenase-2
CREB	cAMP response element-binding protein
CNS	Central nervous system
CUMS	Chronic unpredictable mild stress
DHM	Dihydromyricetin
DNP	Diabetic neuropathic pain
DRG	Dorsal root ganglion
ERK1/2	Extracellular signal-regulated kinases 1 and 2
FMT	Fecal microbiota transplantation
GFAP	Glial fibrillary acidic protein
GPX4	Glutathione peroxidase 4
GSH	Glutathione
GSK-3β	Glycogen synthase kinase-3 beta
HIF	Hypoxia-inducible factor 1-alpha
HO-1	Heme oxygenase-1
HPA	Hypothalamic–pituitary–adrenal
ICH	Intracerebral hemorrhage
IL	Interleukin
iNOS	Inducible nitric oxide synthase
IS	Ischemic stroke
LCN2	Lipocalin-2
LPS	Lipopolysaccharide
MCAO	Middle cerebral artery occlusion
MDD	Major depressive disorder
NF-κB	Nuclear factor kappa B
NLRP3	NLR family pyrin domain containing 3
Nrf2	Nuclear factor erythroid 2-related factor 2
OGD/R	Oxygen-glucose deprivation/reoxygenation
PD	Parkinson’s disease
PI3K	Phosphoinositide 3-kinase
Prx2	Peroxiredoxin 2
ROS	Reactive oxygen species
SAH	Subarachnoid hemorrhage
SCI	Spinal cord injury
SIRT1	Sirtuin 1
SNI	Spared nerve injury
STING	Stimulator of interferon genes
TLR4	Toll-like receptor 4
TNF-α	Tumor necrosis factor alpha
TrkB	Tropomyosin receptor kinase B
VEGFA	Vascular endothelial growth factor A
VEGFR2	Vascular endothelial growth factor receptor 2
