# Supplementary material for: Dihydromyricetin and Neuroinflammation: Implications for CNS Disorders and Therapeutic Development
Source: Int J Mol Sci. 2026 Oct 13;27(20):9080. doi: 10.3390/ijms27209080 (PMC13704991; doi:10.3390/ijms27209080)
Supplement: Supplementary file 1 [file ijms-27-09080-s001.zip › ijms-4586753-supplementary.pdf]

Review

Dihydromyricetin and Neuroinflammation: Implications for CNS Disorders and Therapeutic Development

Supplementary Material

Table S1. ***In vitro*** **DHM exposure conditions and sample sizes in the included studies.**

Disease	Experimental Model(s)	DHM Concentration	Treatment Duration	Sample Size (n)	Design notes	Citation
Alzheimer's Disease	MD2 knock-down BV2 cells	10 or 30 µM	28 hours	n = 3/group	Immortalized cell line (BV2)	[17]
	BV2 Cells	2.5 µg/mL	24 hours	Not Reported	Immortalized cell line (BV2); single concentration; n not reported	[18]
Parkinson's Disease	BV-2 cells	3.25, 6.5, and 12.5 µg/mL	22 hours	Not Reported	Immortalized cell line (BV-2); n not reported	[22]
	human H4 neuroglioma cells	10 µM	16 hours	Not Reported	Immortalized cell line (H4); single concentration; n not reported	[23]
Ischemic Stroke	LPS-stimulated BV2 microglia	0-50 µM (dosed 4 hours pre-injury or simultaneously)	4 or 7 hours	Not Reported	Immortalized cell line (BV2); n not reported	[24]
	HT22 neuronal cells (OGD/R)	10, 25, 50, or 100 µM (simultaneous dosing)	30-32 hours	Not Reported	Immortalized cell line (HT22); n not reported	[24]
	SH-SY5Y cells (OGD/R)	5, 10, 20, 50, and 100 µM (dosed 1 hour pre-injury)	24 hours	Not Reported	Immortalized cell line (SH-SY5Y); n not reported	[25]
	BV2 microglia (OGD/R); HEK293 cells	1, 5, and 10 µM (concurrent with reoxygenation)	24 hours	Not Reported	Immortalized cell lines (BV2, HEK293); n not reported	[26]
Depression	LPS-stimulated BV2 microglia	10, 20, and 40 µM	24 hours	Not Reported	Immortalized cell line (BV2); n not reported	[29]
	primary hippocampal neurons	10, 25, 50 µM	1 hour or 48 hours	Not Reported	Primary cells; n not reported	[31]

	C8-D1A astrocytes (MPP^+)	50 µM (dosed 2 h pre-injury)	2 hours	Not Reported	Immortalized cell lines; n not reported	[32]
	SN4741 dopaminergic neurons (astro-cyte-conditioned medi-um)	50 µM	24 hours	Not Reported	Immortalized cell lines; n not reported	[32]
Subarachnoid hemorrhage (SAH)	primary cortical neurons (oxyhemoglobin-induced injury)	10, 20, and 40 µM	Not Reported	Not Reported	Primary cells; n and duration not reported	[35]
Neuropathic Pain	BV2 cells	10 µM, 100 µM, or 1 mM	24 hours	Not Reported	Immortalized cell line (BV2); n not reported	[37]
	Primary hippocampal astrocytes (high glucose + substance P + corticosterone)	1 µM (simultaneous dosing)	24 hours	Not Reported	Primary cells; single concentration; n not reported	[38]
Spinal Cord Injury	BV-2 microglia (H_2O_2-induced injury)	100 µM (dosed prior to injury)	24 hours	n = 6/group	Immortalized cell line (BV-2); single concentration	[42]

Table S2. ***In vivo*** **DHM dosing conditions and animal characteristics in the included studies.**

Disease	Experimental Model(s)	DHM Dose	Route of Administration	Treatment Duration	Sex	Sample Size (n)	Design notes	Citation
Alzheimer's Disease	APP/PS1 Double Transgenic AD Mice	100 mg/kg and 200 mg/kg	Oral Gavage	30 days	Not Reported	Not Reported	Sex not reported; n not reported	[17]
	APP/PS1 Double Transgenic AD Mice	1 mg/kg	IP Injection	2 weeks and 4 weeks	Not Reported	n = 32	Sex not reported; single dose level	[18]
	Sprague-Dawley rats (intracerebral Aβ1-42 injection)	100 mg/kg or 200 mg/kg	IP Injection	21 days	Male rats	n = 40	Male only	[19]
	Sprague-Dawley rats (ICV Aβ1-42 infusion)	0.5 mg/kg/two days	IP Injection	4 weeks	Male rats	Not Reported	Male only; n not reported; single dose level	[20]
	Obesity-related AD mice	60 mg/kg	Oral Gavage	15 weeks	Male mice	n = 56	Male only; single dose level	[21]
Parkinson's Disease	MPTP/probenecid-treated C57BL/6 mice	100 mg/kg/day	Oral Gavage	8 weeks	Male mice	n = 60	Male only; single dose level	[22]
	BAC-α-syn-GFP transgenic mice	10 mg/kg/day	IP Injection	2 weeks	Not Reported	n = 8/group	Sex not reported; single dose level	[23]
Ischemic Stroke	Male ICR mice (MCAO/reperfusion)	10 or 20 mg/kg (dosed 3 days pre-	IP Injection	7 days	Male mice	n = 10/group	Male only	[24]

		injury and 4 days post-injury)						
	Male Sprague Dawley rats	100 mg/kg (dosed post-injury)	IP Injection	7 days	Male rats	n = 24	Male only; single dose level	[27]
	Male Wistar rats (ischemia/reperfusion)	50 and 100 mg/kg (dosed 10-15 min and 2 hours post-reperfusion)	IP Injection	3 days	Male rats	n = 24 (stage 1); n = 12 (stage 2)	Male only	[28]
Depression	Male C57BL/6 mice (LPS-induced depression)	10 mg/kg, 20 mg/kg, 40 mg/kg	IP Injection	7 days	Male mice	n = 60	Male only	[29]
	Male ICR mice (corticosterone-induced depression)	20 mg/kg	IP Injection	7 days	Male mice	n = 30	Male only; single dose level	[30]
	Male C57BL/6 mice (acute LPS-induced and CUMS depression)	10 or 20 mg/kg	IP Injection	3 days (LPS-induced depression); 7 days (CUMS-induced depression)	Male mice	n = 48	Male only	[31]
	MPTP-induced PD with depression mouse model	10 mg/kg	IP Injection	15 days	Male mice	n = 32	Male only; single dose level	[32]

Social Isolation Induced Anxiety	Male C57BL/6 mice (4-6 weeks social isolation)	2 mg/kg	Oral (agar cube)	2 weeks	Male mice	n = 10-11/group (behav-ioral); n = 5/group (micro-glial/mo-lecular)	Male only; single dose level; n = 5/group for molecular assays	[33]
	Male C57BL/6 mice (4 weeks social isolation)	2 mg/kg	Oral (agar cube)	2 weeks	Male mice	n = 3-4/group	Male only; single dose level; small n (3-4/group)	[34]
Subarachnoid hemorrhage (SAH)	Adult Sprague-Dawley rats (prechiasmatic cistern blood in-jection)	30, 60, or 120 mg/kg (dosed 2 hours post-injury)	Oral Gavage	Single dose	Male rats	n = 158	Male only	[35]
Intracerebral hemorrhage (ICH)	Male C57BL/6 mice (colla-genase-induced ICH)	50 mg/kg, 150 mg/kg, and 300 mg/kg (dosing started 7 days prior to injury)	Oral Gavage	10 days and 21 days	Male mice	n = 72	Male only	[36]
Neuropathic Pain	Male CD1 mice (SNI model)	10 mg/kg (IP route) or 10 µg/10 µL (local injection route)	IP or Local In-jection	6 days	Male mice	n = 10/group	Male only	[37]
	Male Sprague-Dawley rats (di-abetic	30 mg/kg	IP Injection	14 days	Male rats	n = 60	Male only; single dose level	[39]

	neuropathic pain + chronic unpredictable stress)							
	Male C57BL/6 mice (SNI; comorbid neuropathic pain and depression)	20 mg/kg (dosed starting the 2nd week post-injury)	IP Injection	14 days	Male mice	n = 24	Male only; single dose level	[40]
	Male Sprague-Dawley rats (comorbid diabetic neuropathic pain and depression); DRG and spinal cord	30 mg/kg	IP Injection	14 days	Male rats	n = 72	Male only; single dose level	[41]
Spinal Cord Injury	C57BL/6 mice (spinal cord compression)	150 mg/kg (dosed post-injury)	Oral Gavage	28 days	Male mice	n = 75	Male only; single dose level	[42]
	Sprague-Dawley rats (C5 unilateral clip-compression SCI)	100 mg/kg (dosed post-injury)	Oral Gavage	14 days	Male rats	n = 42	Male only; single dose level	[43]
	antibiotic-depleted rats (FMT)	None (recipients received DHM-donor microbiota only)	Oral Gavage	14 days	Male rats	n = 16	Male only; dose not reported	[43]

Epilepsy	Male Sprague-Dawley rats (Pilocarpine-induced status epilepticus)	50 mg/kg or 100 mg/kg (dosed on the 3rd day post-injury)	IP Injection	28 days	Male rats	n = 60	Male only	[44]
